# Microtubules regulate disassembly of epithelial apical junctions

**DOI:** 10.1186/1471-2121-7-12

**Published:** 2006-03-01

**Authors:** Andrei I Ivanov, Ingrid C McCall, Brian Babbin, Stanislav N Samarin, Asma Nusrat, Charles A Parkos

**Affiliations:** 1 Epithelial Pathobiology Research Unit, Department of Pathology and Laboratory Medicine, Emory University, Atlanta, Georgia 30322, USA

## Abstract

**Background:**

Epithelial tight junction (TJ) and adherens junction (AJ) form the apical junctional complex (AJC) which regulates cell-cell adhesion, paracellular permeability and cell polarity. The AJC is anchored on cytoskeletal structures including actin microfilaments and microtubules. Such cytoskeletal interactions are thought to be important for the assembly and remodeling of apical junctions. In the present study, we investigated the role of microtubules in disassembly of the AJC in intestinal epithelial cells using a model of extracellular calcium depletion.

**Results:**

Calcium depletion resulted in disruption and internalization of epithelial TJs and AJs along with reorganization of perijunctional F-actin into contractile rings. Microtubules reorganized into dense plaques positioned inside such F-actin rings. Depolymerization of microtubules with nocodazole prevented junctional disassembly and F-actin ring formation. Stabilization of microtubules with either docetaxel or pacitaxel blocked contraction of F-actin rings and attenuated internalization of junctional proteins into a subapical cytosolic compartment. Likewise, pharmacological inhibition of microtubule motors, kinesins, prevented contraction of F-actin rings and attenuated disassembly of apical junctions. Kinesin-1 was enriched at the AJC in cultured epithelial cells and it also accumulated at epithelial cell-cell contacts in normal human colonic mucosa. Furthermore, immunoprecipitation experiments demonstrated association of kinesin-1 with the E-cadherin-catenin complex.

**Conclusion:**

Our data suggest that microtubules play a role in disassembly of the AJC during calcium depletion by regulating formation of contractile F-actin rings and internalization of AJ/TJ proteins.

## Background

Intercellular junctions are a characteristic morphological feature of differentiated epithelial cell monolayers. They represent several types of multiprotein complexes assembled at distinct positions within the lateral plasma membrane in areas of cell-cell contacts. The tight junction (TJ) is the most apically positioned complex followed by the subjacent adherens junction (AJ). Collectively TJ and AJ are referred to as an apical junctional complex (AJC; [1,2]). In simple epithelia, TJs and AJs function together to create a barrier for paracellular movement of solutes and macromolecules while also playing a crucial role in maintenance of apico-basal cell polarity [3,4]. The integrity and barrier properties of epithelial cell monolayers are ensured by transmembrane TJ and AJ proteins that are engaged in trans-interactions with their partners on the opposing plasma membrane [2,5,6]. Such transmembrane components of TJs include occludin, members of the claudin family, and immunoglobulin-like proteins junctional adhesion molecule (JAM)-A and coxsackie adenovirus receptor [7,8]. Major transmembrane proteins of epithelial AJs include E-cadherin and members of nectin protein family [6,9]. Transmembrane components of apical junctions are clustered and stabilized by an array of intracellular scaffold proteins that create so called TJ and AJ cytosolic plaques. The cytosolic TJ plaque contains many different proteins of which members of the 'zonula occludens' (ZO) protein family are the most extensively characterized [7,8]. The cytosolic AJ plaque include E-cadherin binding partners such as α and β-catenins, and p120 catenin [9,10]. One of the important functions of junctional cytosolic plaques is to provide a link between transmembrane TJ/AJ proteins and the cortical cytosketon [11] allowing efficient transduction of signals from intercellular junctions to the cell interior as well as "inside out signaling" from cytosolic compartments to intercellular contacts [1,12].

An emerging theme of junctional research is centered on understanding mechanisms of AJC disassembly [13-15]. Reversible disruption of epithelial apical junctions is important for embryonic morphogenesis and tissue remodeling [16,17]. Furthermore, disassembly of the AJC plays an important pathophysiological role in the epithelial to mesenchymal transition, a key element in malignant transformation [18]. In addition, disruption of epithelial apical junctions appears to be a common mechanism of host invasion exploited by various bacterial and viral pathogens (reviewed in [15]).

Disassembly of the epithelial AJC appears to be mediated by two major mechanisms. One involves reorganization of perijunctional actin cytoskeleton and another involves endocytosis of junctional proteins. The relationship between these mechanisms is not clear but several recent studies have suggested an important role for F-actin reorganization that results in destabilization of trans-interactions between TJ/AJ proteins of adjacent epithelial cells and triggers AJC internalization [19-21].

Major actin-driven processes such as cell migration, cytokinesis, vesicle and organelle trafficking require the involvement of another component of intracellular cytoskeleton, microtubules [22-24]. Microtubules are filamentous structures created by self-assembly of α /β tubulin heterodimers [25,26]. Similar to F-actin microfilaments, microtubules are polarized by having a fast growing "plus" and a slow growing "minus" ends [27,28]. In columnar epithelial cells, prominent bundles of microtubules align along the lateral plasma membrane. This population of microtubules orient their minus ends toward the cell apex and plus ends toward the cell base [29-31]. In addition, differentiated renal and intestinal epithelial cells exhibit a dense net of microtubules with mixed polarity located at the level of apical junctions [29-31]. Hence, the perijunctional space of differentiated epithelial cells is rich in microtubules. Several recent reports have suggested a relationship between microtubules and apical junctions. For example, formation of AJ-like cell-cell contacts after forced expression of E- and N-cadherin in fibroblasts was shown to stabilize minus ends of microtubules and to promote microtubule polymerization [32]. On the other hand, microtubule depolymerization was shown to disrupt the integrity of TJs and AJs in thyroid and lung epithelial cells [33,34] and to disassemble endothelial AJs [35]. An AJ scaffold protein, p120-catenin, has been reported to associate with microtubules [36,37] and can be transported to intercellular junctions by a microtubule motor, kinesin [38], whereas β-catenin was shown to interact with dynein, another type of microtubule motor [39]. Further evidence for a role of microtubules in regulation of AJC functions was obtained in several disease models. For example, microtubules have been implicated in disruption of endothelial barrier by a tumor necrosis factor-α [40] and thrombin [41], as well as in oxidant-induced increase in permeability of intestinal epithelial monolayers [42]. However, the precise role of microtubules in regulation of epithelial apical junctions remains to be investigated.

Based on the above we hypothesized that microtubules are involved in disruption of apical junctions in simple epithelia. The present study was designed to examine the role of microtubules in disassembly of the AJC by using a classical model of extracellular calcium depletion. We report that microtubules regulate the formation of contractile-F-actin rings and disruption of the AJC in calcium-depleted epithelial cells. Both disintegration of the AJC and reorganization of F-actin are regulated by microtubule turnover (depolymerization/repolymerization) and the activity of microtubule motors, kinesins.

## Results

### Microtubule depolymerization blocked disassembly of the AJC and formation of contractile F-actin rings in calcium-depleted epithelial cells

**Figure 1:**
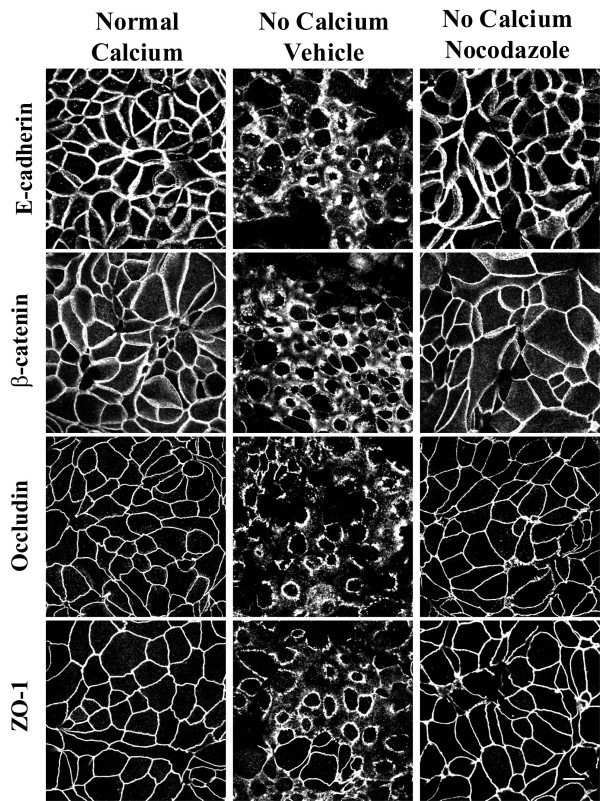
**Depolymerization of microtubules attenuates disassembly of the AJC in calcium-depleted SK-CO-15 cells. **In fully polarized SK-CO-15 cells cultured at normal concentration of extracellular calcium, AJ proteins E-cadherin and β-catenin and TJ proteins occludin and ZO-1 are localized at areas of cell-cell junctions producing a characteristic 'chicken wire' pattern. In vehicle treated cells, 1 h of calcium depletion leads to translocation of junctional proteins from areas of cell-cell contacts into centrally-located ring-like structures. The microtubule-depolymerizing agent nocodazole (30 μM) prevents disassembly of the AJC and cytosolic translocation of junctional proteins. Bar, 10 μm.

The majority of experiments in the present study utilized SK-CO-15 human colonic epithelial cells [43]. These cells rapidly polarize, within 5–7 days creating high-resistance (500–1,000 Ohm × cm^2^) monolayers and are a good model for study of apical junctions in intestinal epithelium [43,44]. To investigate whether disassembly of the AJC in calcium-depleted SK-CO-15 cells is dependent on microtubule integrity, we examined this process in cells where microtubules were depolymerized with nocodazole. At normal (~2 mM) calcium concentrations, epithelial AJ (E-cadherin and β-catenin) and TJ (occludin and ZO-1) proteins were located primarily at the areas of cell-cell contacts creating characteristic "chicken wire" staining patterns (Figure 1). In vehicle-treated SK-CO-15 cells, depletion of extracellular calcium for 1 h caused disruption of AJs and TJs and accumulation of E-cadherin, β-catenin, occludin and ZO-1 in subapical ring-like structures (Figure 1). Treatment of SK-CO-15 cells with nocodazole (30 μM), which effectively eliminated the majority of apical microtubules (see Additional file 1), prevented translocation of all studied AJ/TJ proteins from intercellular junctions to subapical cytosolic rings (Figure 1).

**Figure 2:**
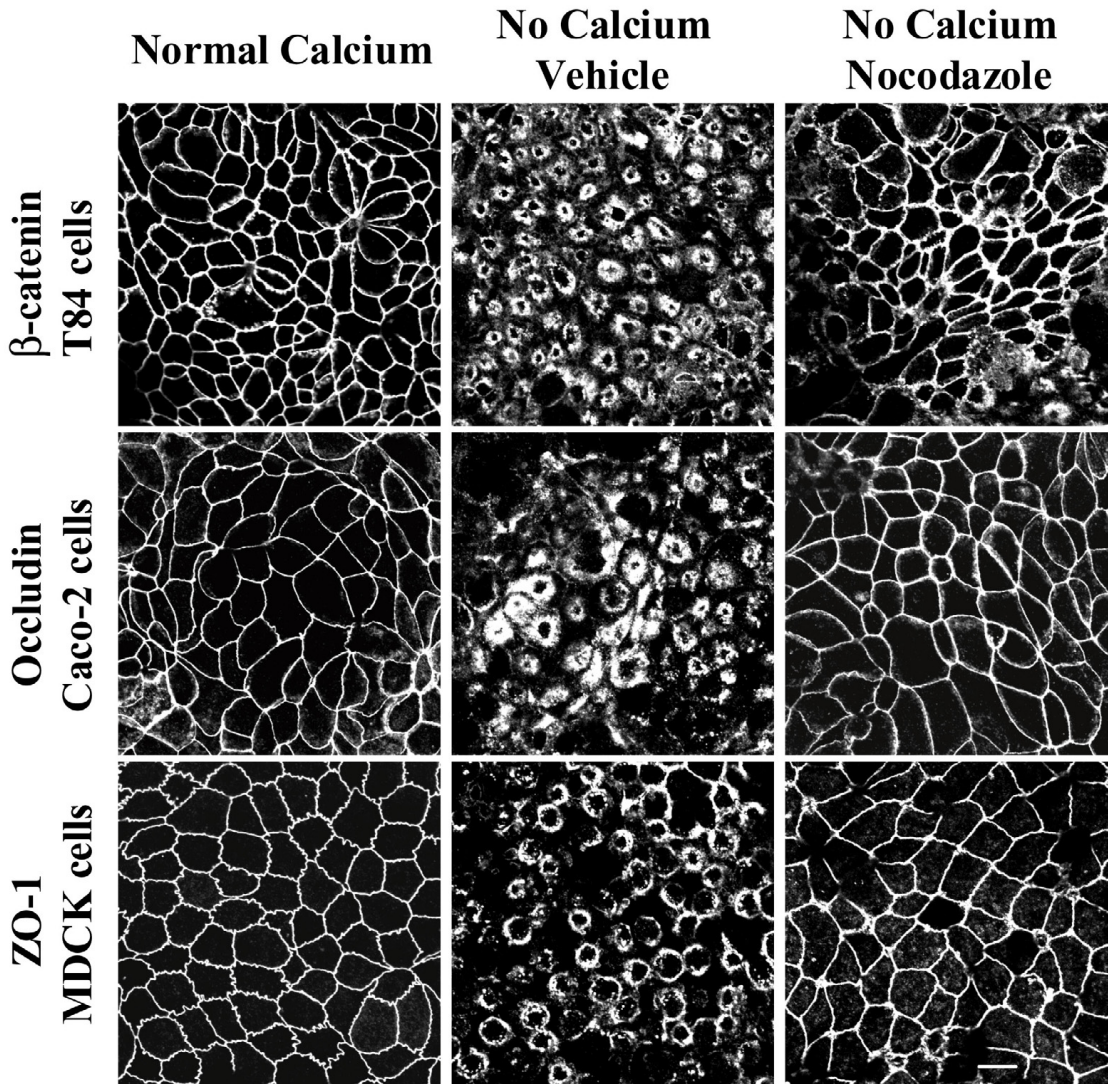
**Depolymerization of microtubules blocks disassembly and internalization of apical junctions in different epithelial cell lines. **Similarly to SK-CO-15 cells, T84, Caco-2 and MDCK cell lines readily respond to 1 h calcium depletion by translocating junctional proteins β-catenin, occludin and ZO-1 from areas of cell-cell contacts into cytosolic ring-like structures. Depolymerization of microtubules with nocodazole (30 μM) inhibits disassembly of the AJC and translocation of junctional proteins in all tested epithelial cell lines. Bar, 10 μm.

To ensure that the microtubule-dependence of AJC disassembly was not unique to SK-CO-15 cells, we investigated whether nocodazole affects disruption of apical junctions in other intestinal (T84 and Caco-2) and renal (MDCK) epithelial cell lines. Immunolabeling for β-catenin, occludin, and ZO-1 in T84, Caco-2 and MDCK cells, respectively, demonstrated similar responses of these cells to 1 h of calcium depletion as manifested by accumulation of junctional proteins in subapical cytosolic rings (Figure 2). Microtubule depolymerization with nocodazole (30 μM) blocked such translocation of AJC proteins into cytosolic ring-like structures in all examined cell lines (Figure 2). These data strongly suggest that microtubule dependence of the AJC disassembly is a common feature of different types of simple epithelia.

**Figure 3:**
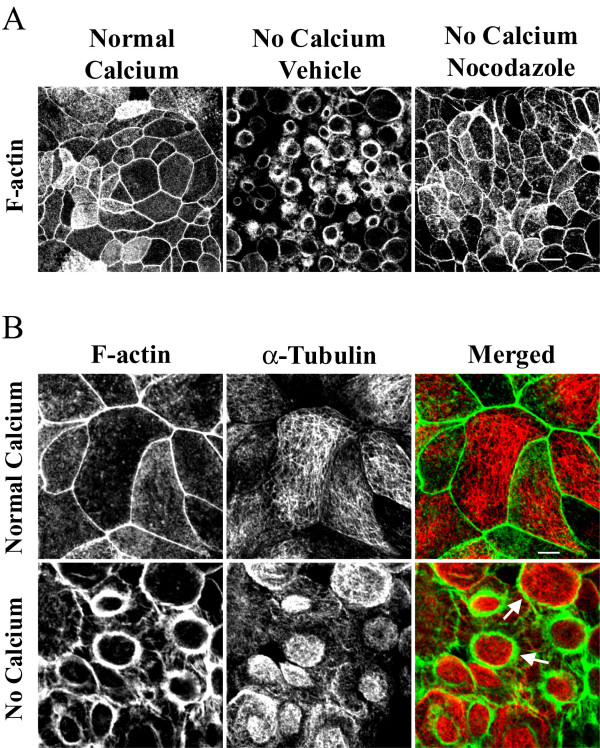
**Microtubules regulate formation of contractile F-actin rings in calcium-depleted epithelial cells. **(A) In confluent SK-CO-15 cells cultured in the normal-calcium medium, apical actin is organized in a perijunctional F-actin belt at the AJC level. In vehicle treated cells, calcium depletion for 1 h resulted in formation of apical contractile F-actin rings, whereas depolymerization of microtubules with nocodazole (30 μM) prevents assembly of F-actin rings. Bar, 10 μm. (B) In SK-CO-15 cells cultured at normal-calcium conditions, apical microtubules (red color) are organized in a loose horizontal meshwork, which does not significantly overlap with F-actin (green color). Calcium depletion results in reorganization of microtubules into dense plaques positioned inside contractile F-actin rings where microtubules partially colocalize with actin microfilaments (arrows). Bar, 5 μm.

Previous studies by several groups including ours [19,45-47] demonstrated that formation of apical contractile F-actin rings plays a central role in driving disassembly of epithelial AJC during calcium depletion. Thus, we hypothesized that microtubules can either control assembly/contraction of F-actin rings or act downstream of actomyosin rings by mediating internalization of junctional proteins from disrupted intercellular contacts. To distinguish between these two mechanisms, we examined the effect of microtubule depolymerization on formation of contractile F-actin rings. SK-CO-15 cells were preincubated with either nocodazole (30 μM) or vehicle followed by 1 h of calcium depletion in the presence of the drug, and the organization of perijunctional actin microfilaments was visualized using a fluorescent derivative of phalloidin. At normal calcium concentration, apical F-actin was organized primarily as a belt encircling the entire cell at the level of the AJC (Figure 2A). In vehicle-treated SK-CO-15 cells, calcium depletion triggered dramatic reorganization of apical actin microfilaments into centrally-located contractile rings (Figure 3A). The formation of such F-actin rings was prevented in nocodazole-treated cells (Figure 3A). Next we performed immunofluorescence labeling for α-tubulin to investigate if calcium depletion causes reorganization of apical microtubules. In polarized SK-CO-15 cells cultured in normal calcium medium, a relatively loose horizontal web of microtubules was observed at the cell apex with a minor fraction extending along the perijunctional F-actin belt (Figure 3B). Calcium depletion caused formation of dense plaques of microtubules positioned within the contractile F-actin rings (Figure 3B). At the periphery of such plaques, microtubules appeared to be in direct contact with actin microfilaments (Figure 3B, arrows). Collectively, these data suggest that calcium depletion triggers orchestrated reorganization of apical F-actin and microtubules and that microtubule integrity is necessary for the assembly of contractile F-actin rings.

### Microtubule stabilization attenuated disassembly of apical junctions and prevented contraction of F-actin rings

**Figure 4:**
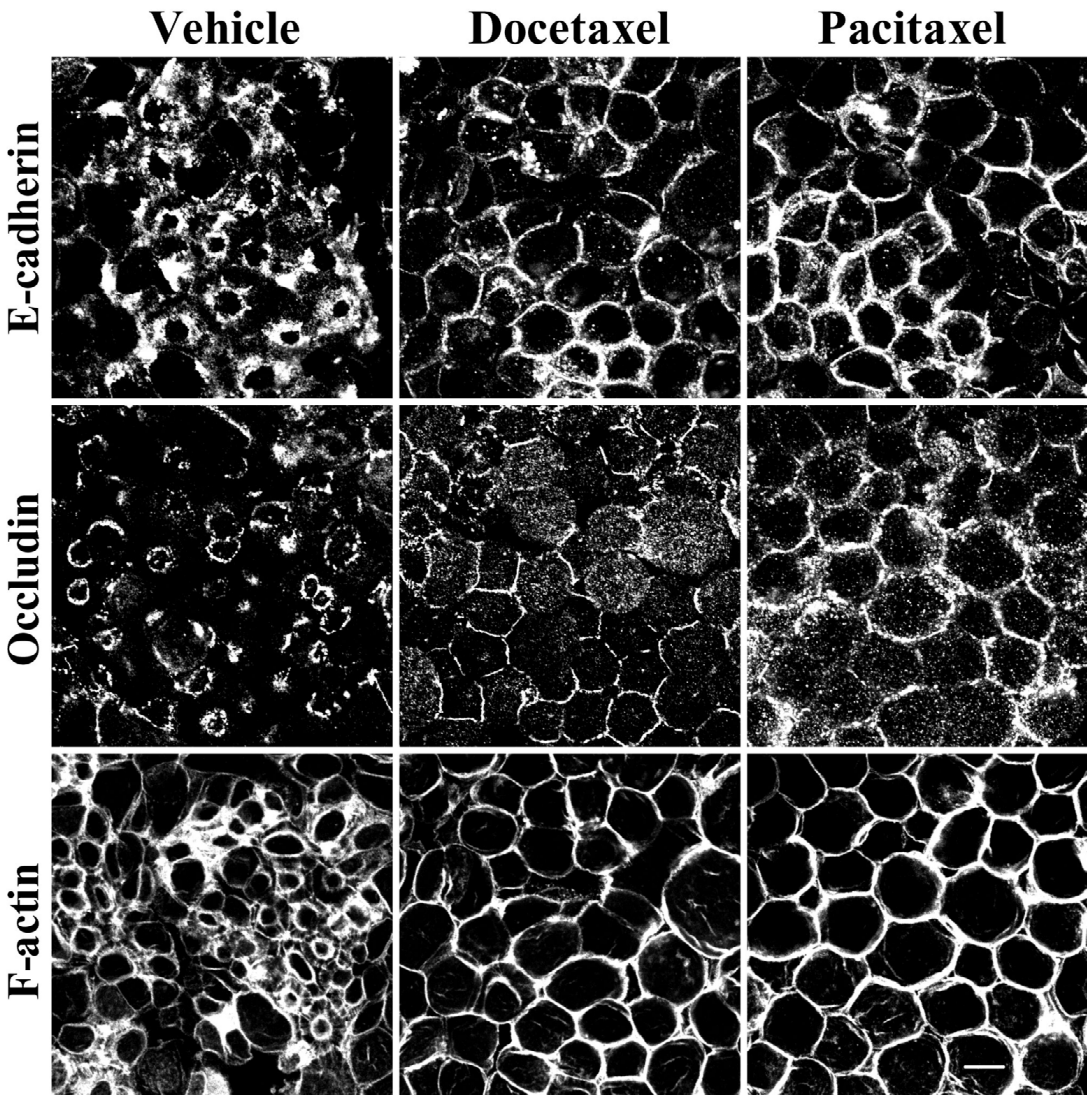
**Stabilization of microtubules attenuates disassembly of apical junctions and inhibits contractility of F-actin rings. **SK-CO-15 cells were incubated for 1 h in calcium-free media in the presence of vehicle or the microtubule-stabilizing drugs docetaxel (10 μM) or pacitaxel (10 μM). Note that microtubule stabilization attenuates disassembly of the AJC and translocation of E-cadherin and occludin into the subapical cytosolic compartment and also blocks contraction of F-actin rings. Bar, 10 μm.

Reorganization of microtubules during calcium-depletion is likely to involve their movement and spatial reorientation. This process can occur via two different mechanisms that were attributed to two populations of microtubules with different turnover rates. Redistribution of dynamic (rapidly turning over) microtubules is controlled by polymerization and depolymerization at opposite ends of their filaments (treadmilling mechanism), whereas translocation/reorientation of stable (slowly turning over) microtubules can be driven by microtubule motors [27,28,31,48]. To determine whether microtubule turnover is essential for the formation of contractile F-actin rings and disassembly of the AJC in calcium-depleted epithelial cells, we used two microtubule stabilizing drugs, docetaxel and pacitaxel. These agents have been reported to suppress microtubule depolymerization and promote their polymerization [49-51]. We rationalized that inhibition or attenuation of AJC disassembly by microtubule stabilization would indicate a role for dynamic microtubules in this process. SK-CO-15 cells were pretreated with either docetaxel or pacitaxel (both at 10 μM) followed by 1 h of calcium depletion in the presence of the same concentrations of the drugs. Such exposure of cell monolayers to pacitaxel (Additional file 1) or docetaxel (data not shown) significantly increased the density of apical microtubules, thus confirming the microtubule-stabilizing action of these drugs. In calcium-depleted SK-CO-15 cells, both agents attenuated disassembly of the AJC and translocation of E-cadherin and occludin from cell-cell contacts into cytosolic ring-like structures (Figure 4). These effects appeared to be transient, since after 2 h of calcium depletion, the majority of E-cadherin and occludin disappeared from apical junctions even in microtubule-stabilized cells (data not shown). In addition to attenuation of AJC disassembly, docetaxel or pacitaxel prevented the formation of apical contractile F-actin rings (Figure 4). These findings strongly suggest that dynamic microtubules regulate disassembly and internalization of TJs and AJs as well as contraction of perijunctional F-actin microfilaments in calcium-depleted epithelial cells.

**Figure 5:**
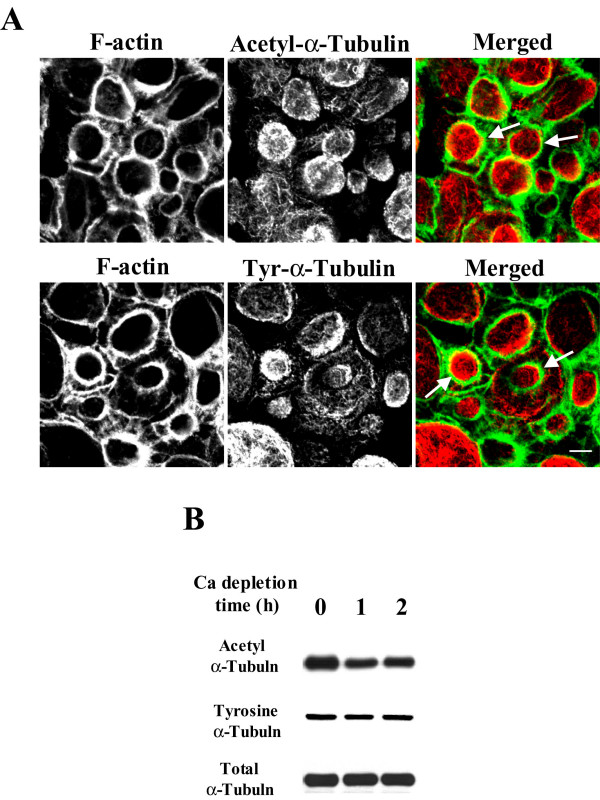
**Calcium depletion causes reorganization of acetylated and tyrosinated apical microtubules and decreases the amount of acetylated microtubules. **(A) Acetylated (Acetyl) and tyrosinated (Tyr) microtubules (red color) are accumulated inside of contractile F-actin rings (green color) after 1 h of calcium depletion of SK-CO-15 cells. Bar, 5 μm. (B) Representative Western blots demonstrate significant change in expression of acetylated but not total or tyrosinated α-tubulin in SK-CO-15 cells after 1 and 2 h of calcium depletion relatively to a non-depleted control.

Given the important role for microtubule dynamics in the AJC disassembly, we sought to investigate if calcium depletion alters balance between stable and dynamic microtubules in epithelial cells. Stability of microtubules in living cells is controlled by several mechanisms; the best characterized involves posttranslational modifications of α-tubulin [52,53]. A common posttranslational modification occurs via enzymatic removal and readdition of a tyrosine residue at the C-terminal end of α-tubulin that produces detyrosinated and tyrosinated (Tyr) tubulin, respectively [52,54]. Detyrosinated and Tyr-tubulin comprise two distinct microtubule populations *in vivo *where the former is considered stable and the later is highly dynamic [52,53]. Another posttranslational modification of α-tubulin that stabilizes microtubules involves acetylation of a lysine residue at position 40 in the amino-terminus [53,54]. We examined distribution and expression of stable and dynamic microtubules in control and calcium-depleted SK-CO-15 cells using two monoclonal antibodies specific for acetylated and tyrosinated α-tubulin. Immunofluorescence labeling and confocal microscopy demonstrated accumulation of both acetylated (stable) and tyrosinated (unstable) microtubules inside contractile F-actin rings in calcium-depleted cells (Figure 5A, arrows). Furthermore, Western blot analyses demonstrated a significant decrease in the amount of acetylated but not tyrosinated or total α-tubulin in SK-CO-15 cells after 1 and 2 h of calcium depletion (Figure 5B), thus indicating decreased microtubule stability. This observation confirmed our pharmacological data suggesting that dynamic microtubules are involved in the formation of contractile F-actin rings and disassembly of the epithelial AJC.

### Inhibition of kinesin motors attenuated reorganization of apical F-actin and disassembly of the AJC

**Figure 6:**
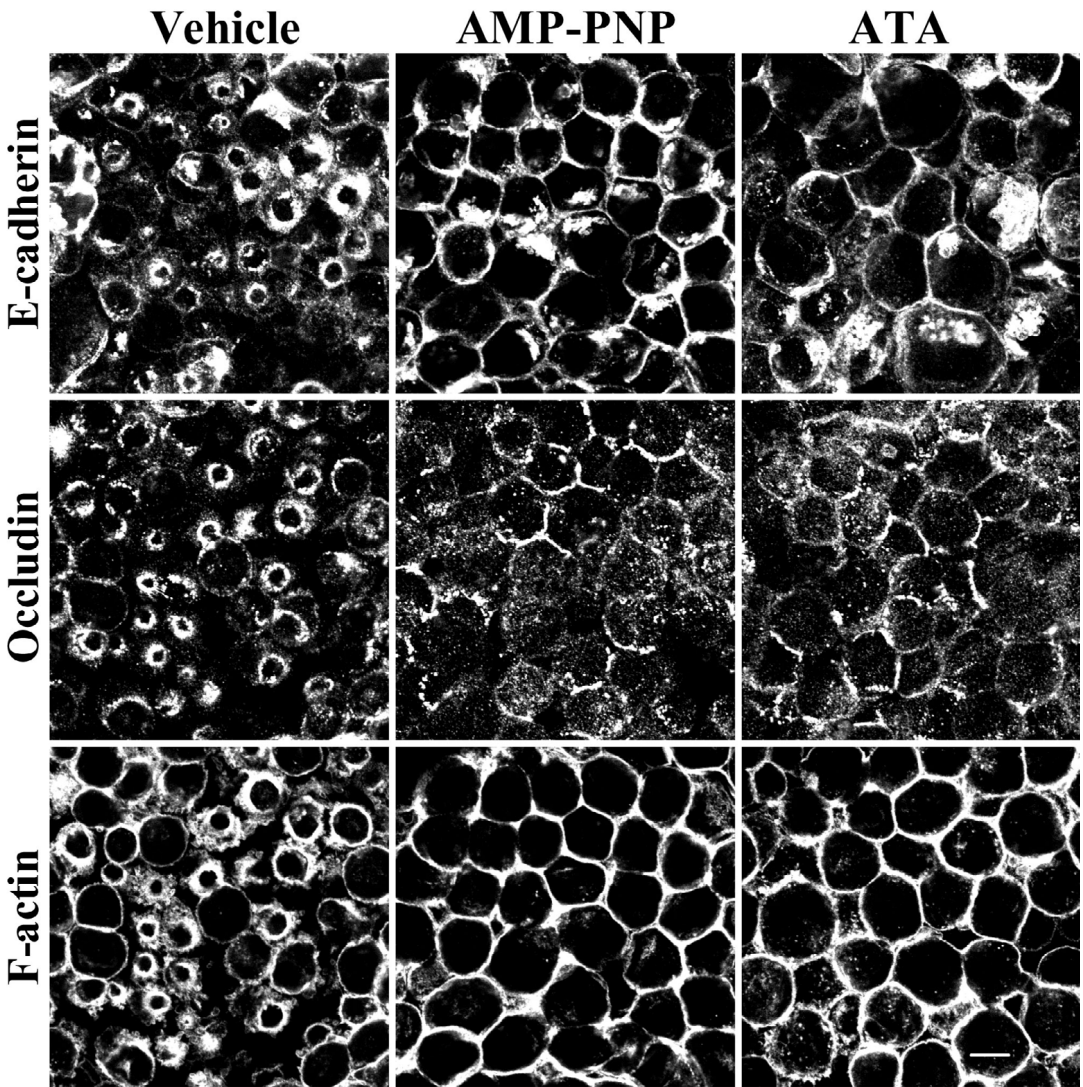
**Kinesin microtubule motors mediate disassembly/internalization of AJs and TJs and regulate contraction of F-actin rings. **SK-CO-15 cells were incubated for 1 h in calcium-free media in the presence of either vehicle or kinesin inhibitors 5' -adenylylimidodiphosphate (AMP-PNP, 500 μM) or aurintricarboxylic acid (ATA, 50 μM). Note that inhibition of kinesin motor activity blocks contraction of F-actin rings and translocation of E-cadherin and occludin into subapical cytosolic rings. Bar, 10 μm.

Reorganization of microtubules as well as vesicle trafficking along them in epithelial cells are known to be regulated by microtubule motors [31,48,55]. These proteins mediate unidirectional movement of cargo towards plus or minus ends and thus are categorized as "plus end" or "minus end" motors [48,55]. Members of the kinesin protein family encompass plus end microtubule motors whereas dynein represents a minus end motor [55,56]. Since both kinesins [37,38] and dynein [39] have been shown to interact with and mediate intracellular trafficking of AJ plaque proteins, we reasoned that the polarity of perijunctional microtubules would dictate which type of microtubule motor is involved in disassembly and internalization of the AJC. There is evidence to suggest that microtubules in polarized epithelial cells are organized in such a fashion that their minus ends are enriched at the cell apex close to apical junctions [29,30]. Hence, one would predict that internalization of junctional proteins would be directed toward microtubule plus ends and should thus be mediated by kinesin motors. We tested this hypothesis by investigating effects of two pharmacological inhibitors of kinesins viz., adenylylimidodiphosphate (AMP-PNP, [57,58]) and aurintricarboxylic acid (ATA, [59,60]), on AJC disassembly during calcium depletion. SK-CO-15 cells were pretreated with either AMP-PNP (500 μM) or ATA (50 μM) or vehicle followed by 1 h calcium depletion in the presence of the inhibitors. Both AMP-PNP and ATA attenuated translocation of E-cadherin and occludin from apical junctions to the subapical cytosolic compartment and blocked contraction of F-actin rings (Figure 6). Since these pharmacological inhibitors are effective against all kinesin classes, we next attempted to identify which class of kinesins may mediate the formation of contractile F-actin rings and internalization of AJC proteins during calcium depletion. Although approximately 45 kinesin genes have been recognized in the mammalian genome [61], the two likely candidate regulators of AJC disassembly and internalization are kinesin-1 and kinesin-2. In particular, kinesin-1 has been reported to mediate trafficking of the AJ proteins N-cadherin [62] and p120 catenin [37,38] and, in MDCK cells, it has been shown to localize in areas of cell-cell contacts [37]. Kinesin-2, on the other hand, has been reported to interact with the PAR-3-PAR-6-atypical PKC polarity complex in neurons [63] and has been implicated in the formation of the apical plasma membrane domain in polarized kidney epithelial cells [64,65].

**Figure 7:**
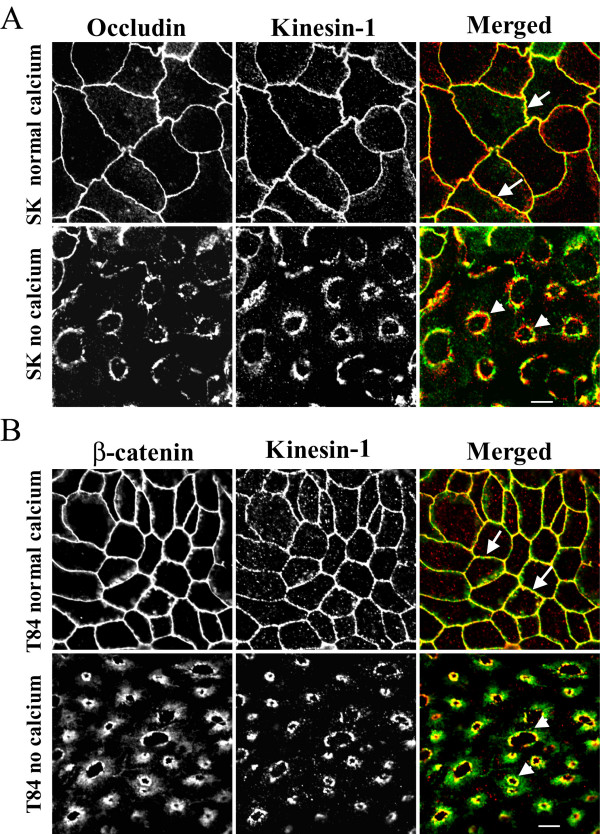
**Kinesin-1 is localized at intact and internalized apical junctions in colonic epithelial cells. **In confluent SK-CO-15 (A) and T84 (B) epithelial cell monolayers cultured at high-calcium conditions, kinesin-1 (red color) colocalizes with the TJ protein occludin and the AJ protein β-catenin (arrows). In calcium-depleted cells, kinesin-1 colocalizes with internalized junctional proteins (arrowheads). Bar, 5 μm.

**Figure 8:**
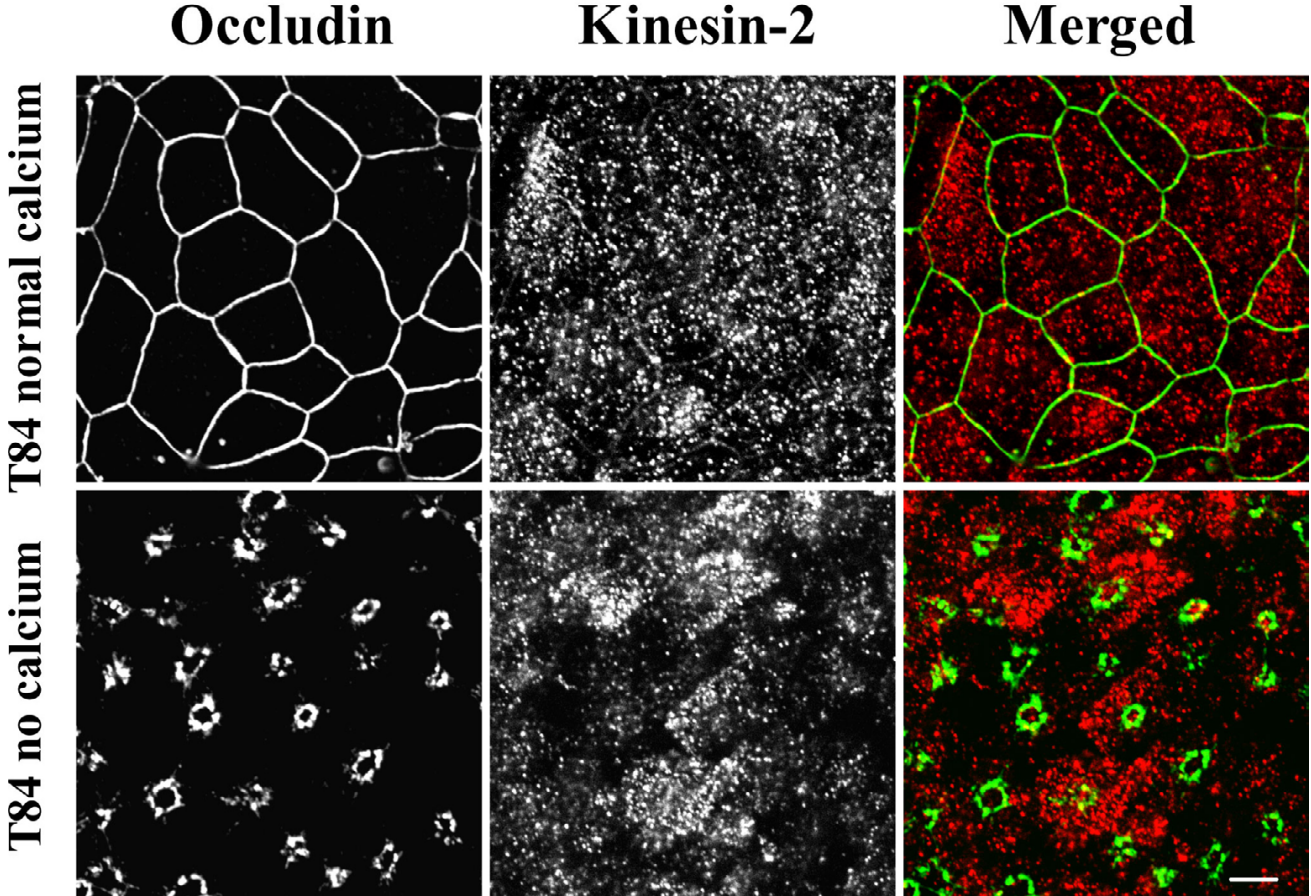
**Kinesin-2 is not localized at intact or internalized epithelial apical junctions. **In polarized T84 epithelial cells monolayers cultured under normal conditions, or in the calcium-depleted conditions, kinesin-2 (red color) is diffusely distributed in cytosol and does not colocalize with the TJ protein occludin (green color). Bar, 5 μm.

**Figure 9:**
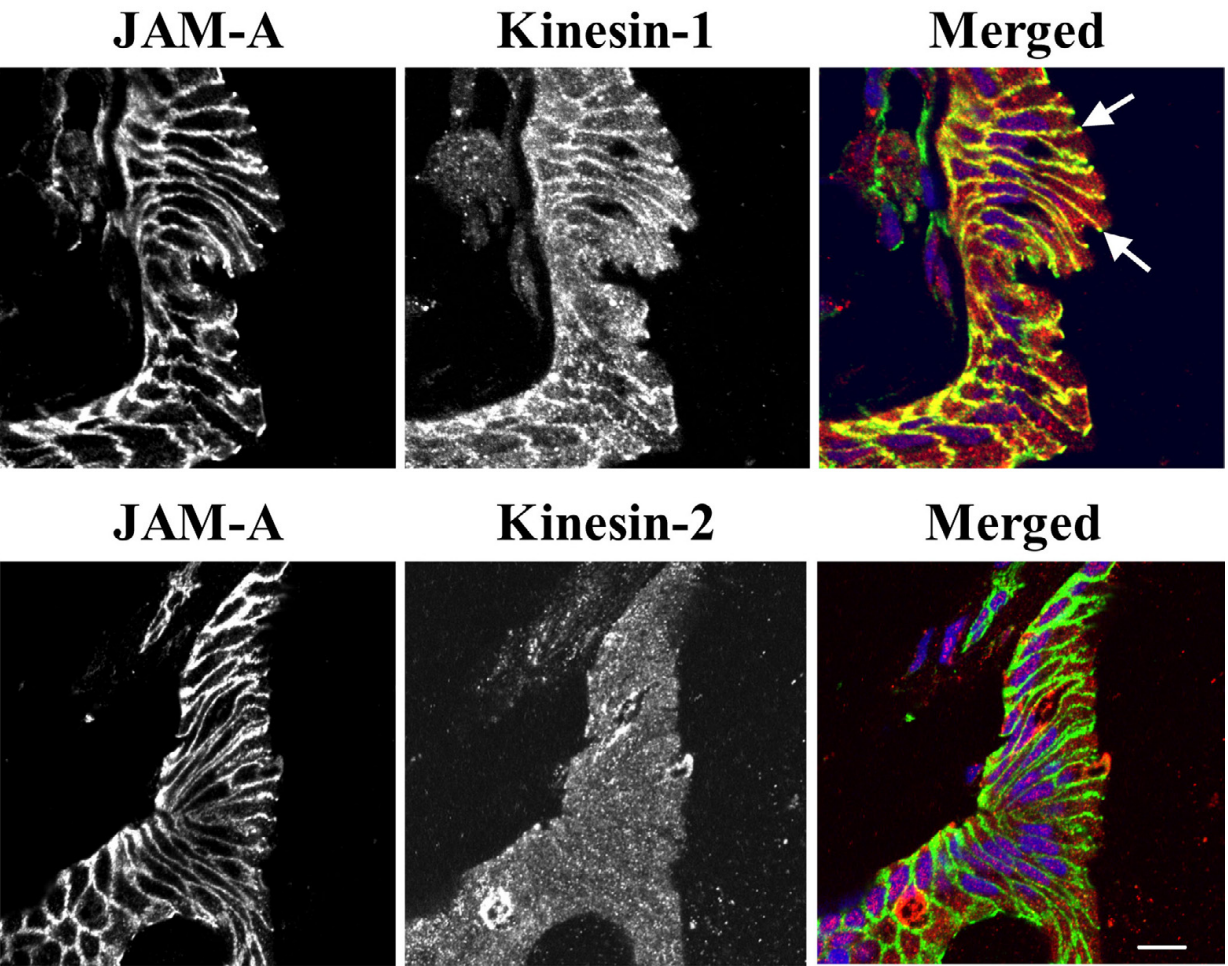
**Kinesin-1 is enriched at the areas of cell-cell contacts in normal human colonic mucosa. **Cross-sections of normal human colonic mucosa reveal a significant pool of kinesin-1 (red color) localized at the areas of cell-cell contacts where it colocalizes with the AJC protein, JAM-A (arrows). By contrast, kinesin-2 (red) labeling demonstrates diffuse intracellular distribution of this protein without significant colocalization with JAM-A (green) at intercellular junctions. Bar, 10 μm.

We thus analyzed the distribution of kinesins 1 and 2 in control and calcium-depleted colonic epithelial cells using anti-kinesin heavy-chains-specific monoclonal antibodies. Kinesin-1 appeared to be selectively enriched at the AJC in polarized SK-CO-15 and T84 cells where it colocalized with occludin and β-catenin (Figure 7A, B, arrows). In calcium-depleted epithelial cells, kinesin-1 redistributed from areas of cell-cell contacts along with junctional proteins and colocalized with internalized occludin and β-catenin in the subapical cytosolic compartment (Figure 7A, B, arrowheads). In contrast, kinesin-2 staining was dot-like throughout the cell and did not colocalize with AJC proteins in control and calcium-depleted T84 (Figure 8) and SK-CO-15 (data not shown) cells. These findings suggest that the kinesin-1 motor may play a role in regulating endocytosis and intracellular trafficking of TJ/AJ proteins during calcium depletion. To test physiological relevance of these in vitro kinesin immunolabeling data, we examined localization of kinesin-1 in normal human colonic mucosa. We observed significant enrichment of kinesin-1 at the areas of lateral cell-cell contacts between differentiated colonocytes at the mucosal surface (Figure 9). This labeling pattern was indistinguishable from that of the known AJC protein JAM-A, and both kinesin-1 and JAM-A significantly colocalized at intercellular junctions (Figure 9, arrows). In contrast, kinesin-2 demonstrated diffuse intracellular distribution in normal colonic mucosa and did not accumulate in areas of cell-cell contact, nor did it colocalize with JAM-A (Figure 9).

**Figure 10:**
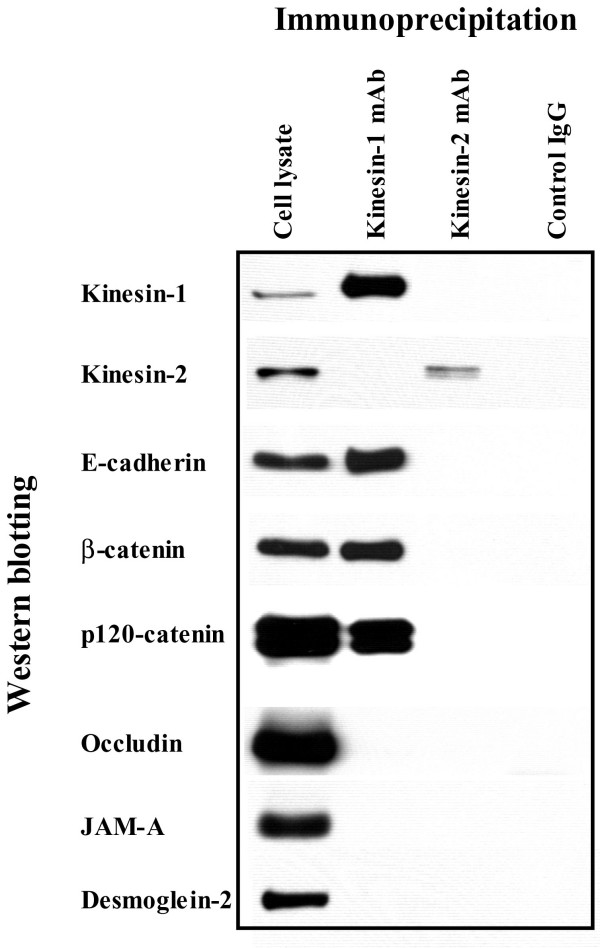
**Association of kinesin-1 with AJ proteins. **T84 cell lysates were immunoprecipitated with either anti-kinesin-1 heavy chain antibody, anti-kinesin-2 heavy chain antibody, or control mouse IgG and immunoblotted for different junctional proteins as described in the Methods section. Kinesin-1 co-precipitates with AJ proteins E-cadherin, β-catenin and p120 catenin, but not with TJ proteins occludin and JAM-A or desmosomal component desmoglein-2. No co-pecipitation of any tested junctional protein with either kinesin-2 or control IgG is seen.

Given our intracellular localization data for kinesin-1 in Figures 7 and 9, we next examined if kinesin-1 was physically associated with AJC proteins. Immunoprecipitates obtained from intestinal epithelial cells using the anti-kinesin-1 heavy chain antibody were analyzed for the associated junctional proteins by Western blotting. We found that kinesin-1 immunoprecipitates from T84 cells contained readily detectable amounts of the AJ proteins E-cadherin, β-catenin and p120 catenin (Figure 10). By contrast, no co-immunoprecipitation of the TJ proteins occludin and JAM-A, or the desmosomal protein desmoglein-2 was detected. Similar co-precipitation of kinesin-1 with AJ proteins was detected in SK-CO-15 cells (data not shown). The anti-kinesin-2 heavy chain antibody did not co-precipitate any junctional proteins under these experimental conditions (Figure 10). Likewise, a control mouse IgG did not co-immunoprecipitate AJC proteins from SK-CO-15 (Figure 10) or T84 (data not shown) cell lysates. Collectively, these immunoprecipitation data suggest specific association of kinesin-1 heavy chain with the E-cadherin-catenin complex in intestinal epithelial cells.

## Discussion

### Microtubules regulate disassembly of epithelial apical junctions by controlling formation of contractile F-actin rings

In the present study, we report a novel mechanism that regulates disassembly of the epithelial AJC by using a classical tool of junctional research, depletion of extracellular calcium. It is generally believed that disintegration of epithelial monolayers during calcium depletion is initiated by disruption of trans-dimers of E-cadherin, the only calcium-binding protein in apical junctions [66,67]. We recently observed that such perturbation of E-cadherin-mediated adhesion in human intestinal epithelial cells triggers the selective early formation of contractile apical actomyosin rings without obvious effects on general cell shape and apico-basal cell polarity [19]. It is likely that contraction of apical F-actin rings in calcium-depleted cells provides mechanical force to overcome trans-interactions between various junctional proteins in two adjacent membranes and to separate intercellular adhesions [19,46,47]. This activates endocytosis of AJC components, in turn accelerating cell separation [20]. In the present study, we found that actomyosin contraction alone is not sufficient to disrupt the AJC in calcium-depleted epithelial cells and that microtubules are critical for this process. Two lines of evidence support this conclusion. First, pharmacological modulation (disruption or stabilization) of microtubules themselves or inhibition of microtubule motors kinesins prevented or attenuated disassembly of the AJC (Figures 1, 2, 4 and 6) and the formation of contractile F-actin rings (Figures 3A, 4 and 6). Second, in calcium-depleted cells, apical microtubules reorganized into dense plaques positioned inside contractile actomyosin rings (Figure 3B) and kinesin-1 colocalized with intact and internalized apical junctions (Figures 7, 9).

Our finding that depolymerization of microtubules prevents the formation of contractile F-actin rings (Figure 3) suggests that microtubules act upstream of the actomyosin reorganization during calcium depletion. It is likely that microtubules either transduce signals from disrupted AJs to the apical actomyosin cytoskeleton or regulate reorganization of actin microfilaments. Indeed, physical interactions of microtubules with epithelial AJs (via p120 catenin [11]) and with F-actin (via coronin, IQGAP, the dynein/dynactin complex, unconventional myosins, etc., [23,24]) are well documented.

How microtubules can regulate the formation and contraction of apical actomyosin rings in calcium-depleted cells remains enigmatic. In a previous study [19] we proposed that these apical F-actin rings resemble contractile actomyosin structures involved in a variety of physiological processes such as cytokinesis and wound closure. Our present observation of microtubule-dependent biogenesis of actomyosin rings during calcium depletion reinforces this idea, since microtubules have been shown to modulate organization of contractile apparatus in cleavage furrows between dividing mammalian cells [68,69] or around wounds made in *Xenopus *oocytes [70]. These interesting mechanistic parallels strongly suggest that the same signaling cascades/molecular complexes may be involved in cytoskeleton-dependent AJC disassembly in calcium-depleted epithelial cells and in other contractile processes such as furrow ingression and wound closure. Several recent studies suggest a molecular mechanism by which microtubules regulate actomyosin contractility during cytokinesis [71,72] and wound closure [73]. This mechanism involves members of the Rho family of small GTPases. Microtubules have been shown to regulate localized activity of Rho GTPases by binding to and accumulating Rho GTP exchange factors and/or Rho GTPase-activating proteins [24], which are positive and negative regulators of Rho GTPase activity, respectively. We speculate, therefore, that Rho GTPases may also link reorganization of microtubules and the actomyosin cytoskeleton in calcium-depleted cells. Indeed, Rho GTPases have been directly implicated in regulation of assembly and integrity of intercellular junctions [11,74] and we have preliminary data suggesting an important role of Rho-associated kinase in disassembly of AJC during calcium depletion (data not shown).

### Role of microtubule dynamics in disassembly of the AJC and biogenesis of contractile F-actin rings

Our results indicate that turnover of microtubules is important for disassembly/internalization of apical junctions and biogenesis of contractile F-actin rings. Turnover of microtubules can occur either by dynamic instability (alternating phases of growth and shrinkage at the plus end) or treadmilling (growth of the plus end and shortening at the minus end) [26-28]. Depending on the rate of the above processes, microtubules can be classified as either dynamic or stable. The former is turned over within 5 min, whereas the later remain intact for several hours [75,76]. In living cells, these two populations of microtubules appear to be spatially and functionally segregated as has been shown for neurite outgrowth [52], migration of fibroblasts [77] and intracellular vesicle trafficking in hepatic cells [78]. Here, we report that microtubule stabilization with docetaxel or pacitaxel significantly attenuated disruption of TJs and AJs (Figures 4) and contraction of apical F-actin rings in calcium-depleted epithelial cells (Figure 4). These effects of microtubule stabilization appear to be transient since prolonged calcium depletion resulted in AJ and TJ disruption even in microtubule-stabilized cells. However in docetaxel or pacitaxel-treated cells, internalized AJC proteins were diffusely distributed in the cytosol and did not accumulate in subapical ring-like structures (data not shown). These observations suggest a specific role of microtubule turnover in regulation of intracellular trafficking of AJC proteins. Interestingly, dynamic microtubules may also be involved in disruption of endothelial barrier caused by thrombin and transforming growth factor since microtubule stabilization with pacitaxel was shown to completely block such stimulus-induced disassembly of AJs and reorganization of F-actin in cultured endothelial cells [41,79]. These results are consistent with published data on the role of microtubule turnover in biogenesis of contractile rings during cytokinesis and wound healing where stabilization of microtubules inhibited contraction-driven cleavage furrow ingression [80] and wound closure [70].

Importantly, disassembly of both endothelial and epithelial junctions is accompanied by changes in the stability of microtubules. Indeed, either thrombin or cytokine-induced disintegration of endothelial AJs was paralleled by the decrease in the amount of acetylated α-tubulin [41,79], which is a marker for stable microtubules. Similarly, AJC disassembly in calcium-depleted SK-CO-15 cells resulted in decreased acetylation of α-tubulin (Figure 5B), suggesting increased instability of microtubule [53,54]. In contrast, the amount of Tyr-tubulin in epithelial cells was not affected by calcium depletion (Figure 5B), thus illustrating a preserved balance between tyrosinated and detyrosinated microtubules. Collectively, these data suggest that deacetylation rather then tyrosination represents a specific mechanism for increased microtubule dynamics that may regulate disassembly of intercellular junctions in epithelial and endothelial cell monolayers.

### Microtubule motors, kinesins, are involved in disassembly of epithelial AJC

We observed that two structurally different inhibitors of kinesin motor activity, AMP-PNP and ATA, blocked contraction of apical F-actin rings and attenuated disruption /internalization of apical junctions in calcium-depleted cells (Figure 6). This is the first functional data implicating kinesin motors in down-regulation of cell-cell adhesion. Furthermore, our findings suggest that two major mechanisms of microtubule reorganization including microtubule turnover and kinesin-driven motility cooperate in disassembly of the AJC. Interestingly, we previously found similar cooperation between actin turnover and myosin II-mediated reorganization of actin microfilaments during disruption and reestablishment of epithelial apical junctions [19,81].

Our immunolabeling/confocal microscopy data strongly implicated kinesin-1 in the biogenesis of contractile F-actin rings and/or intracellular trafficking of internalized AJC proteins in calcium-depleted cells. Kinesin-1 (formally known as conventional kinesin or KIF5, [82]) is a plus end microtubule motor functioning as a tetramer of two heavy chains and two light chains [56,61]. The kinesin-1 heavy chain contains a motor domain that binds to microtubules and has intrinsic ATPase activity [83]. We found that kinesin-1 heavy chain was specifically enriched at the AJC in polarized SK-CO-15 and T84 epithelial cells where it colocalized with TJ and AJ proteins (Figure 7). Furthermore, in normal human colonic mucosa, kinesin-1 was also enriched at the areas of cell-cell contacts (Figure 9). Specific localization of kinesin-1 at the epithelial AJC is a novel finding. Indeed, previous studies either detected accumulation of kinesin-1 at non-specified lateral contacts in MDCK cells [37], or failed to observe enrichment of kinesin-1 in intercellular junctions of PtK2 epithelial cells [84]. Importantly, we found not only localization of kinesin-1 at the epithelial AJC, but also co-precipitation of this motor protein with AJ proteins, E-cadherin, β-catenin and p120 catenin (Figure 10). These data are in agreement with previous reports of association of kinesin-1 with p120 catenin in fibroblasts [37,38,62]. However, our study provides the first evidence that this microtubule motor may bind to the assembled E-cadherin-catenin complex in polarized epithelial cells. During calcium depletion, kinesin-1 redistributed from the disrupted AJC into the subapical cytosolic compartment where it colocalized with internalized TJ /AJ components (Figure 7). This finding, together with our pharmacological data (Figure 6), strongly suggests the involvement of kinesin-1 in intracellular trafficking of AJC proteins along microtubule tracks. In contrast, our data do not support the role of kinesin-2 in reorganization of apical junctions and epithelial cytoskeleton in calcium-depleted cells (Figures 8, 9, 10).

## Conclusion

The present study describes a novel mechanism that regulates disassembly of epithelial apical junctions during extracellular calcium depletion. This mechanism involves microtubule-mediated formation of contractile actomyosin rings that break cell-cell contacts as well as microtubule-dependent transport of internalized junctional proteins. Dynamic reorganization of microtubules and activity of kinesin motors are essential for these processes. Furthermore, our data highlight potential roles of kinesin-1 in cytoskeletal reorganization and trafficking of AJC proteins in calcium-depleted epithelial cells. Such microtubule-dependent disassembly of the AJC may represent a common mechanism that underlies physiological remodeling of apical junctions during normal epithelial morphogenesis and mediates disruption of epithelial barriers by pathogenic and inflammatory stimuli.

## Methods

### Antibodies and other reagents

The following primary polyclonal (pAb) and monoclonal (mAb) antibodies were used to detect TJ, AJ, and microtubule proteins by immunoflurescence labeling and Western blotting: anti-occludin, ZO-1, E-cadherin mAbs, anti-occludin and JAM-A pAbs (Zymed Laboratories, San Francisco, CA); anti-β-catenin pAb, anti-total α-tubulin, acetylated-tubulin and tyrosinated-tubulin mAbs (Sigma-Aldrich, St. Louis, MO), anti-kinesin heavy chain (H1) mAb (Chemicon International, Temecula, CA); anti-kinesin-2 (K2.4) mAb (Covance, Berkley, CA). Alexa-488-conjugated phalloidin, as well as Alexa-488 or Alexa-568 conjugated donkey anti-rabbit and goat anti-mouse secondary antibodies were obtained from Molecular Probes (Eugene, OR); horseradish peroxidase-conjugated goat anti-rabbit and anti-mouse secondary antibodies were obtained from Jackson Immunoresearch Laboratories (West Grove, PA).

Docetaxel was generously provided by Sanifi-Aventis. Nocodazole, pacitaxel, 5' -adenylylimidodiphosphate, aurintricarboxylic acid and other reagents of the highest analytical grade were obtained from Sigma.

### Cell culture

SK-CO-15, a transformed human colonic epithelial cell line, was a gift from Dr. Enrique Rodriguez-Boulan, Weill Medical College of Cornell University, New York. SK-CO-15, Caco-2 and Madin Darby Canine Kidney (MDCK) epithelial cells (both from the American Type Culture Collection, Manassas, VA) were grown in Dulbecco's modified Eagle's medium supplemented with 10% fetal bovine serum, 2 mM l-glutamine, 15 mM HEPES, 1% nonessential amino acids, 40 μg/ml penicillin and 100 μg/ml streptomycin, pH 7.4. T84 cells (American Type Culture Collection) were cultured in a 1:1 mixture of Dulbecco's modified Eagle medium and Ham's F-12 medium supplemented with 10 mM HEPES, 14 mM NaHCO_3_, 40 μg/ml penicillin, 100 μg/ml streptomycin, 5% newborn calf serum, pH 7.4. For all experiments, epithelial cells were grown for 6–10 days on collagen-coated, permeable polycarbonate filters, 0.4 μm pore size (Costar, Cambridge, MA). Filters with a surface area of 0.33 and 5 cm^2 ^were used for immunocytochemical and biochemical experiments respectively.

### Calcium depletion and pharmacological modulation of apical junction disassembly

To deplete extracellular Ca^2+^, confluent epithelial monolayers were washed twice with calcium-free Eagle's minimum essential medium for suspension culture (S-MEM, Sigma) supplemented with 2 mM EGTA, 10 mM HEPES, and either 10% dialyzed fetal bovine serum (for SK-CO-15, MDCK and Caco-2) or 5% dialyzed newborn calf serum (for T84 cells) and were incubated in S-MEM for indicated times at 37°C [19, 85]. For microtubule depolymerization, cells were preincubated in media containing normal levels of calcium and 30 μM nocodazole for 60 min at 37°C and then for another 60 min at 4°C. After this preincubation, cells were incubated for 60 min at 37°C in the S-MEM containing the same concentration of nocodazole. This temperature-switching protocol was devised to promote disassembly of all microtubules including nocodazole-resistant, cold sensitive ones [86]. For microtubule stabilization or kinesin inhibition, cells were preincubated with drugs in normal calcium media for 60 min at 37°C followed by 60 min incubation in S-MEM containing the same concentration of inhibitors. Stock solutions of water-insoluble inhibitors were prepared in DMSO and diluted in cell culture media immediately before each experiment. The final concentration of DMSO was 0.1%; the same concentration of the vehicle was included in appropriate controls.

### Immunofluorescence labeling

Calcium-depleted epithelial monolayers were rinsed twice with ice-cold calcium- and magnesium-free Hanks balanced salt solution containing 10 mM HEPES, whereas control monolayers were rinsed with HEPES-buffered Hanks balanced salt solution containing calcium and magnesium (HBSS^+^). Cells were fixed/permeabilized in absolute methanol (-20°C for 20 min) followed by blocking in HBSS^+ ^containing 1% bovine serum albumin (blocking buffer) for 60 min at room temperature and incubation for 60 min with primary antibodies in blocking buffer. Cell monolayers were then washed, incubated for 60 min with Alexa dye-conjugated secondary antibodies followed by rinsing and mounting on slides with ProLong Antifade medium (Molecular Probes). For fluorescent labeling of microtubules or F-actin, monolayers were fixed for 15 min in 3.7% paraformaldehyde (PFA), permeabilized for 10 min with 0.5% Triton X-100 (TX-100) and sequentially stained with primary anti-tubulin and Alexa dye-conjugated secondary antibodies, whereas F-actin was labeled with Alexa-conjugated phalloidin. For tissue labeling, 5 μm frozen tissue sections of normal human colon obtained from discarded surgical resection specimens at the Emory University Hospital were mounted on glass coverslips, air-dried and fixed in 100% ethanol (-20°C for 20 min) and immunolabeled as described above. Stained cell monolayers and tissue sections were examined using a Zeiss LSM510 laser scanning confocal microscope (Zeiss Microimaging Inc., Thornwood, NY) coupled to a Zeiss 100 M axiovert and 63× or 100× Pan-Apochromat oil lenses. Fluorescent dyes were imaged sequentially in frame-interlace mode to eliminate cross talk between channels. Images shown are representative of at least 3 experiments, with multiple images taken per slide.

### Immunoblotting

Cells were homogenized in a RIPA lysis buffer (20 mM Tris, 50 mM NaCl, 2 mM EDTA, 2 mM EGTA, 1% sodium deoxycholate, 1% TX-100, and 0.1% SDS, pH 7.4), containing a proteinase inhibitor cocktail (1:100, Sigma) and phosphatase inhibitor cocktails 1 and 2 (both at 1:200, Sigma). Lysates were then cleared by centrifugation (20 min at 14, 000 × g) diluted with 2× SDS sample buffer and boiled. Polyacrylamide gel electrophoresis and Western blotting were conducted by standard methods with 10–20 μg protein per lane. The results shown are representative immunoblots of three independent experiments.

### Immunoprecipitation

Confluent monolayers of T84 and SK-CO-15 cells were harvested into an immunoprecipitation buffer (50 mM PIPES, 50 mM HEPES, 1 mM EDTA, 2 mM MgSO_4_, 1% TX-100, and 0.5% Igepal, pH 7.0), supplemented with proteinase inhibitor cocktail and phosphatase inhibitor cocktails 1 and 2. Cells were homogenized, centrifuged and supernatants were precleared with Protein G-coupled Sepharose beads (Amersham Biosciences, Buckinghamshire, UK) for 60 min at 4°C. Precleared lysates (500 μl) were then incubated overnight at 4°C with 5 μg of either anti-kinesin-1 heavy chain mAb (clone H1; Chemicon), anti-kinesin-2 heavy chain mAb (clone K2.4; Covance) or control mouse IgG (Jackson Laboratories). Immunocomplexes were recovered by incubation with Protein G-Sepharose beads for 3 h at 4°C with constant rotation. The beads were pelleted and washed four times (10 min per wash at 4°C) with the immunoprecipitation buffer. Beads were then boiled for 5 min in 80 μl of 2× SDS sample buffer and pelleted by centrifugation. Equal volumes of supernatants (20 μl) were loaded into polyacrylamide gels and analyzed by electrophoresis and Western blotting as described above. T84 or SK-CO-15 total cell lysates loaded at 15 μg of total protein per lane were used as a control.

## List of abbreviations

AJ, adherens junction; AJC, apical junctional complex; AMP-PNP, 5' -adenylylimidodiphosphate; ATA, aurintricarboxylic acid; MDCK, Madin Darby Canine Kidney; PFA, paraformaldehyde; TJ, tight junction; TX-100, Triton X-100; ZO-1, zonula occludens-1

## Authors' contributions

AII conceived of the study, carried out immunofluorescence labeling and immunoprecipitation experiments and drafted the manuscript; ICM carried out Western blot analysis; BB conducted immunofluorescence labeling of tissue sections; SNS participated in confocal microscopy data acquisition and prepared figures for the manuscript; AN and CAP participated in design and coordination of the study and helped to draft the manuscript.

## Supplementary Material

Additional File 1 **Effects of nocodazole and pacitaxel on apical microtubules in colonic epithelial cells. **Polarized monolayers of SK-CO-15 cells were treated for 2 h with either nocodazole (30 μM) or pacitaxel (10 μM) or vehicle, and microtubules and apical junctions were visualized using antibodies against α-tubulin and β-catenin respectively. As can be seen, the microtubule-depolymerizing agent nocodazole causes disappearance of the tubulin filament meshwork at the level of the AJC, whereas microtubule-stabilizing drug pacitaxel dramatically increases the density of apical microtubules. Click here for file

## References

[B1] Matter K, Balda MS (2003). Signalling to and from tight junctions. Nat Rev Mol Cell Biol.

[B2] Tsukita S, Furuse M, Itoh M (2001). Multifunctional strands in tight junctions. Nat Rev Mol Cell Biol.

[B3] Madara JL (1998). Regulation of the movement of solutes across tight junctions. Annu Rev Physiol.

[B4] Nelson WJ (2003). Adaptation of core mechanisms to generate cell polarity. Nature.

[B5] Yap AS, Brieher WM, Gumbiner BM (1997). Molecular and functional analysis of cadherin-based adherens junctions. Annu Rev Cell Dev Biol.

[B6] Takai Y, Nakanishi H (2003). Nectin and afadin: novel organizers of intercellular junctions. J Cell Sci.

[B7] D'Atri F, Citi S (2002). Molecular complexity of vertebrate tight junctions (Review). Mol Membr Biol.

[B8] Gonzalez-Mariscal L, Betanzos A, Nava P, Jaramillo BE (2003). Tight junction proteins. Prog Biophys Mol Biol.

[B9] Gooding JM, Yap KL, Ikura M (2004). The cadherin-catenin complex as a focal point of cell adhesion and signalling: new insights from three-dimensional structures. Bioessays.

[B10] Pokutta S, Weis WI (2002). The cytoplasmic face of cell contact sites. Curr Opin Struct Biol.

[B11] Perez-Moreno M, Jamora C, Fuchs E (2003). Sticky business: orchestrating cellular signals at adherens junctions. Cell.

[B12] Erez N, Bershadsky A, Geiger B (2005). Signaling from adherens-type junctions. Eur J Cell Biol.

[B13] Bryant DM, Stow JL (2004). The ins and outs of E-cadherin trafficking. Trends Cell Biol.

[B14] D'Souza-Schorey C (2005). Disassembling adherens junctions: breaking up is hard to do. Trends Cell Biol.

[B15] Ivanov AI, Nusrat A, Parkos CA (2005). Endocytosis of the apical junctional complex: mechanisms and possible roles in regulation of epithelial barriers. Bioessays.

[B16] Jarrett O, Stow JL, Yap AS, Key B (2002). Dynamin-dependent endocytosis is necessary for convergent-extension movements in Xenopus animal cap explants. Int J Dev Biol.

[B17] Miller JR, McClay DR (1997). Characterization of the role of cadherin in regulating cell adhesion during sea urchin development. Dev Biol.

[B18] Palacios F, Tushir JS, Fujita Y, D'Souza-Schorey C (2005). Lysosomal targeting of E-cadherin: a unique mechanism for the down-regulation of cell-cell adhesion during epithelial to mesenchymal transitions. Mol Cell Biol.

[B19] Ivanov AI, McCall IC, Parkos CA, Nusrat A (2004). Role for actin filament turnover and a myosin II motor in cytoskeleton-driven disassembly of the epithelial apical junctional complex. Mol Biol Cell.

[B20] Izumi G, Sakisaka T, Baba T, Tanaka S, Morimoto K, Takai Y (2004). Endocytosis of E-cadherin regulated by Rac and Cdc42 small G proteins through IQGAP1 and actin filaments. J Cell Biol.

[B21] Le TL, Joseph SR, Yap AS, Stow JL (2002). Protein kinase C regulates endocytosis and recycling of E-cadherin. Am J Physiol Cell Physiol.

[B22] Brown SS (1999). Cooperation between microtubule- and actin-based motor proteins. Annu Rev Cell Dev Biol.

[B23] Goode BL, Drubin DG, Barnes G (2000). Functional cooperation between the microtubule and actin cytoskeletons. Curr Opin Cell Biol.

[B24] Rodriguez OC, Schaefer AW, Mandato CA, Forscher P, Bement WM, Waterman-Storer CM (2003). Conserved microtubule-actin interactions in cell movement and morphogenesis. Nat Cell Biol.

[B25] Nogales E (2001). Structural insight into microtubule function. Annu Rev Biophys Biomol Struct.

[B26] Amos LA (2004). Microtubule structure and its stabilisation. Org Biomol Chem.

[B27] Howard J, Hyman AA (2003). Dynamics and mechanics of the microtubule plus end. Nature.

[B28] Valiron O, Caudron N, Job D (2001). Microtubule dynamics. Cell Mol Life Sci.

[B29] Bacallao R, Antony C, Dotti C, Karsenti E, Stelzer EH, Simons K (1989). The subcellular organization of Madin-Darby canine kidney cells during the formation of a polarized epithelium. J Cell Biol.

[B30] Meads T, Schroer TA (1995). Polarity and nucleation of microtubules in polarized epithelial cells. Cell Motil Cytoskeleton.

[B31] Musch A (2004). Microtubule organization and function in epithelial cells. Traffic.

[B32] Chausovsky A, Bershadsky AD, Borisy GG (2000). Cadherin-mediated regulation of microtubule dynamics. Nat Cell Biol.

[B33] Waterman-Storer CM, Salmon WC, Salmon ED (2000). Feedback interactions between cell-cell adherens junctions and cytoskeletal dynamics in newt lung epithelial cells. Mol Biol Cell.

[B34] Yap AS, Stevenson BR, Abel KC, Cragoe EJJ, Manley SW (1995). Microtubule integrity is necessary for the epithelial barrier function of cultured thyroid cell monolayers. Exp Cell Res.

[B35] Birukova AA, Smurova K, Birukov KG, Usatyuk P, Liu F, Kaibuchi K, Ricks-Cord A, Natarajan V, Alieva I, Garcia JG, Verin AD (2004). Microtubule disassembly induces cytoskeletal remodeling and lung vascular barrier dysfunction: role of Rho-dependent mechanisms. J Cell Physiol.

[B36] Franz CM, Ridley AJ (2004). p120 catenin associates with microtubules: inverse relationship between microtubule binding and Rho GTPase regulation. J Biol Chem.

[B37] Yanagisawa M, Kaverina IN, Wang A, Fujita Y, Reynolds AB, Anastasiadis PZ (2004). A novel interaction between kinesin and p120 modulates p120 localization and function. J Biol Chem.

[B38] Chen X, Kojima S, Borisy GG, Green KJ (2003). p120 catenin associates with kinesin and facilitates the transport of cadherin-catenin complexes to intercellular junctions. J Cell Biol.

[B39] Ligon LA, Karki S, Tokito M, Holzbaur EL (2001). Dynein binds to beta-catenin and may tether microtubules at adherens junctions. Nat Cell Biol.

[B40] Petrache I, Birukova A, Ramirez SI, Garcia JG, Verin AD (2003). The role of the microtubules in tumor necrosis factor-alpha-induced endothelial cell permeability. Am J Respir Cell Mol Biol.

[B41] Birukova AA, Birukov KG, Smurova K, Adyshev D, Kaibuchi K, Alieva I, Garcia JG, Verin AD (2004). Novel role of microtubules in thrombin-induced endothelial barrier dysfunction. Faseb J.

[B42] Banan A, Choudhary S, Zhang Y, Fields JZ, Keshavarzian A (2000). Oxidant-induced intestinal barrier disruption and its prevention by growth factors in a human colonic cell line: role of the microtubule cytoskeleton. Free Radic Biol Med.

[B43] Le Bivic A, Sambuy Y, Mostov K, Rodriguez-Boulan E (1990). Vectorial targeting of an endogenous apical membrane sialoglycoprotein and uvomorulin in MDCK cells. J Cell Biol.

[B44] Mandell KJ, Babbin BA, Nusrat A, Parkos CA (2005). Junctional adhesion molecule 1 regulates epithelial cell morphology through effects on beta1 integrins and Rap1 activity. J Biol Chem.

[B45] Castillo AM, Lagunes R, Urban M, Frixione E, Meza I (1998). Myosin II-actin interaction in MDCK cells: role in cell shape changes in response to Ca2+ variations. J Muscle Res Cell Motil.

[B46] Ma TY, Tran D, Hoa N, Nguyen D, Merryfield M, Tarnawski A (2000). Mechanism of extracellular calcium regulation of intestinal epithelial tight junction permeability: role of cytoskeletal involvement. Microsc Res Tech.

[B47] Volberg T, Geiger B, Kartenbeck J, Franke WW (1986). Changes in membrane-microfilament interaction in intercellular adherens junctions upon removal of extracellular Ca2+ ions. J Cell Biol.

[B48] McNiven MA, Marlowe KJ (1999). Contributions of molecular motor enzymes to vesicle-based protein transport in gastrointestinal epithelial cells. Gastroenterology.

[B49] Diaz JF, Andreu JM (1993). Assembly of purified GDP-tubulin into microtubules induced by taxol and taxotere: reversibility, ligand stoichiometry, and competition. Biochemistry.

[B50] Ringel I, Horwitz SB (1991). Studies with RP 56976 (taxotere): a semisynthetic analogue of taxol. J Natl Cancer Inst.

[B51] Yvon AM, Wadsworth P, Jordan MA (1999). Taxol suppresses dynamics of individual microtubules in living human tumor cells. Mol Biol Cell.

[B52] Bulinski JC, Gundersen GG (1991). Stabilization of post-translational modification of microtubules during cellular morphogenesis. Bioessays.

[B53] Westermann S, Weber K (2003). Post-translational modifications regulate microtubule function. Nat Rev Mol Cell Biol.

[B54] MacRae TH (1997). Tubulin post-translational modifications--enzymes and their mechanisms of action. Eur J Biochem.

[B55] Vale RD (2003). The molecular motor toolbox for intracellular transport. Cell.

[B56] Hirokawa N, Noda Y, Okada Y (1998). Kinesin and dynein superfamily proteins in organelle transport and cell division. Curr Opin Cell Biol.

[B57] Bananis E, Murray JW, Stockert RJ, Satir P, Wolkoff AW (2000). Microtubule and motor-dependent endocytic vesicle sorting in vitro. J Cell Biol.

[B58] Kapoor TM, Mitchison TJ (1999). Allele-specific activators and inhibitors for kinesin. Proc Natl Acad Sci U S A.

[B59] Guignot J, Caron E, Beuzon C, Bucci C, Kagan J, Roy C, Holden DW (2004). Microtubule motors control membrane dynamics of Salmonella-containing vacuoles. J Cell Sci.

[B60] Hopkins SC, Vale RD, Kuntz ID (2000). Inhibitors of kinesin activity from structure-based computer screening. Biochemistry.

[B61] Hirokawa N, Takemura R (2004). Kinesin superfamily proteins and their various functions and dynamics. Exp Cell Res.

[B62] Mary S, Charrasse S, Meriane M, Comunale F, Travo P, Blangy A, Gauthier-Rouviere C (2002). Biogenesis of N-cadherin-dependent cell-cell contacts in living fibroblasts is a microtubule-dependent kinesin-driven mechanism. Mol Biol Cell.

[B63] Nishimura T, Kato K, Yamaguchi T, Fukata Y, Ohno S, Kaibuchi K (2004). Role of the PAR-3-KIF3 complex in the establishment of neuronal polarity. Nat Cell Biol.

[B64] Fan S, Hurd TW, Liu CJ, Straight SW, Weimbs T, Hurd EA, Domino SE, Margolis B (2004). Polarity proteins control ciliogenesis via kinesin motor interactions. Curr Biol.

[B65] Lin F, Hiesberger T, Cordes K, Sinclair AM, Goldstein LS, Somlo S, Igarashi P (2003). Kidney-specific inactivation of the KIF3A subunit of kinesin-II inhibits renal ciliogenesis and produces polycystic kidney disease. Proc Natl Acad Sci U S A.

[B66] Koch AW, Bozic D, Pertz O, Engel J (1999). Homophilic adhesion by cadherins. Curr Opin Struct Biol.

[B67] Troyanovsky S (2005). Cadherin dimers in cell-cell adhesion. Eur J Cell Biol.

[B68] Fishkind DJ, Silverman JD, Wang YL (1996). Function of spindle microtubules in directing cortical movement and actin filament organization in dividing cultured cells. J Cell Sci.

[B69] Wheatley SP, Wang Y (1996). Midzone microtubule bundles are continuously required for cytokinesis in cultured epithelial cells. J Cell Biol.

[B70] Mandato CA, Bement WM (2003). Actomyosin transports microtubules and microtubules control actomyosin recruitment during Xenopus oocyte wound healing. Curr Biol.

[B71] D'Avino PP, Savoian MS, Glover DM (2005). Cleavage furrow formation and ingression during animal cytokinesis: a microtubule legacy. J Cell Sci.

[B72] Glotzer M (2005). The molecular requirements for cytokinesis. Science.

[B73] Benink HA, Bement WM (2005). Concentric zones of active RhoA and Cdc42 around single cell wounds. J Cell Biol.

[B74] Braga VM (1999). Small GTPases and regulation of cadherin dependent cell-cell adhesion. Mol Pathol.

[B75] Webster DR, Gundersen GG, Bulinski JC, Borisy GG (1987). Differential turnover of tyrosinated and detyrosinated microtubules. Proc Natl Acad Sci U S A.

[B76] Webster DR, Borisy GG (1989). Microtubules are acetylated in domains that turn over slowly. J Cell Sci.

[B77] Nagasaki T, Liao G, Gundersen GG (1994). Isolated plasma membranes induce the loss of oriented detyrosinated microtubules and other contact inhibition-like responses in migrating NRK cells. J Cell Sci.

[B78] Pous C, Chabin K, Drechou A, Barbot L, Phung-Koskas T, Settegrana C, Bourguet-Kondracki ML, Maurice M, Cassio D, Guyot M, Durand G (1998). Functional specialization of stable and dynamic microtubules in protein traffic in WIF-B cells. J Cell Biol.

[B79] Birukova AA, Birukov KG, Adyshev D, Usatyuk P, Natarajan V, Garcia JG, Verin AD (2005). Involvement of microtubules and Rho pathway in TGF-beta1-induced lung vascular barrier dysfunction. J Cell Physiol.

[B80] Shannon KB, Canman JC, Moree CB, Tirnauer JS, Salmon ED (2005). Taxol-stabilized Microtubules Can Position the Cytokinetic Furrow in Mammalian Cells. Mol Biol Cell.

[B81] Ivanov AI, Hunt D, Utech M, Nusrat A, Parkos CA (2005). Differential roles for actin polymerization and myosin II motor in assembly of the epithelial junctional complex. Mol Biol Cell.

[B82] Lawrence CJ, Dawe RK, Christie KR, Cleveland DW, Dawson SC, Endow SA, Goldstein LS, Goodson HV, Hirokawa N, Howard J, Malmberg RL, McIntosh JR, Miki H, Mitchison TJ, Okada Y, Reddy AS, Saxton WM, Schliwa M, Scholey JM, Vale RD, Walczak CE, Wordeman L (2004). A standardized kinesin nomenclature. J Cell Biol.

[B83] Cross RA (2004). The kinetic mechanism of kinesin. Trends Biochem Sci.

[B84] Ligon LA, Tokito M, Finklestein JM, Grossman FE, Holzbaur EL (2004). A direct interaction between cytoplasmic dynein and kinesin I may coordinate motor activity. J Biol Chem.

[B85] Ivanov AI, Nusrat A, Parkos CA (2004). Endocytosis of epithelial apical junctional proteins by a clathrin-mediated pathway into a unique storage compartment. Mol Biol Cell.

[B86] Kreitzer G, Schmoranzer J, Low SH, Li X, Gan Y, Weimbs T, Simon SM, Rodriguez-Boulan E (2003). Three-dimensional analysis of post-Golgi carrier exocytosis in epithelial cells. Nat Cell Biol.

