# Supplementary figures and images for: Microtubules regulate disassembly of epithelial apical junctions
Source: BMC Cell Biol. 2006 Mar 1;7:12. doi: 10.1186/1471-2121-7-12 (PMC1444913; doi:10.1186/1471-2121-7-12)

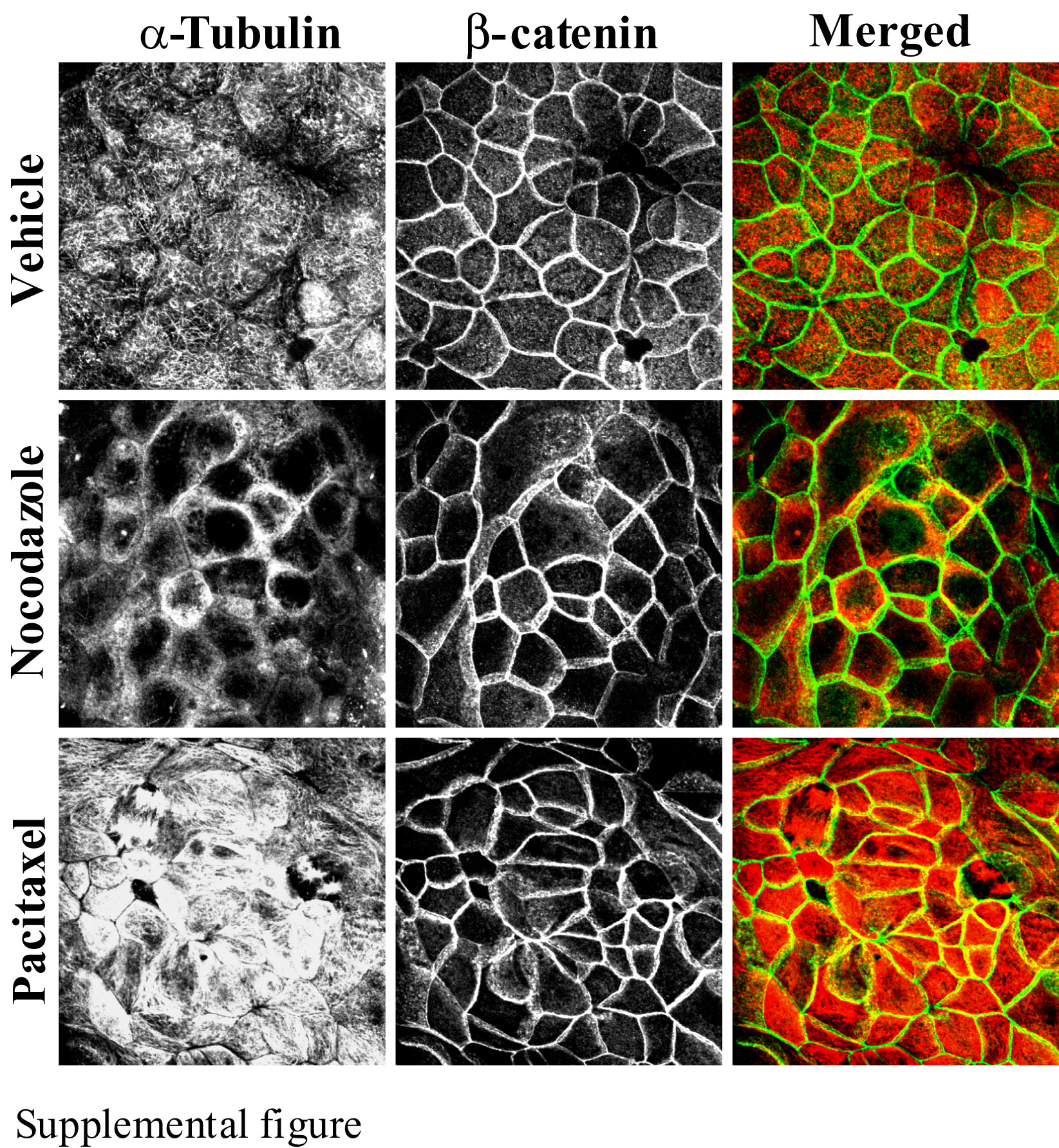

Supplement: Additional File 1 — Effects of nocodazole and pacitaxel on apical microtubules in colonic epithelial cells. Polarized monolayers of SK-CO-15 cells were treated for 2 h with either nocodazole (30 μM) or pacitaxel (10 μM) or vehicle, and microtubules and apical junctions were visualized using antibodies against α-tubulin and β-catenin respectively. As can be seen, the microtubule-depolymerizing agent nocodazole causes disappearance of the tubulin filament meshwork at the level of the AJC, whereas microtubule-stabilizing drug pacitaxel dramatically increases the density of apical microtubules. [file 1471-2121-7-12-S1.jpeg]
